# Cloning, Characterization and Analysis of *cat* and *ben* Genes from the Phenol Degrading Halophilic Bacterium *Halomonas organivorans*

**DOI:** 10.1371/journal.pone.0021049

**Published:** 2011-06-10

**Authors:** Maria de Lourdes Moreno, Cristina Sánchez-Porro, Francine Piubeli, Luciana Frias, María Teresa García, Encarnación Mellado

**Affiliations:** 1 Department of Microbiology and Parasitology, University of Sevilla, Sevilla, Spain; 2 Department of Food Science, University of Campinas, Sao Paulo, Brazil; University of Groningen, Netherlands

## Abstract

**Background:**

Extensive use of phenolic compounds in industry has resulted in the generation of saline wastewaters that produce significant environmental contamination; however, little information is available on the degradation of phenolic compounds in saline conditions. *Halomonas organivorans* G-16.1 (CECT 5995^T^) is a moderately halophilic bacterium that we isolated in a previous work from saline environments of South Spain by enrichment for growth in different pollutants, including phenolic compounds. PCR amplification with degenerate primers revealed the presence of genes encoding ring-cleaving enzymes of the β-ketoadipate pathway for aromatic catabolism in *H. organivorans*.

**Findings:**

The gene cluster *cat*RBCA, involved in catechol degradation, was isolated from *H. organivorans*. The genes *cat*A, *cat*B, *cat*C and the divergently transcribed *cat*R code for catechol 1,2-dioxygenase (1,2-CTD), *cis,cis*-muconate cycloisomerase, muconolactone delta-isomerase and a LysR-type transcriptional regulator, respectively. The benzoate catabolic genes (*ben*A and *ben*B) are located flanking the *cat* genes. The expression of *cat* and *ben* genes by phenol and benzoic acid was shown by RT-PCR analysis. The induction of *cat*A gene by phenol and benzoic acid was also probed by the measurement of 1,2-CTD activity in *H. organivorans* growth in presence of these inducers. 16S rRNA and *cat*A gene-based phylogenies were established among different degrading bacteria showing no phylogenetic correlation between both genes.

**Conclusions/Significance:**

In this work, we isolated and determined the sequence of a gene cluster from a moderately halophilic bacterium encoding *ortho*-pathway genes involved in the catabolic metabolism of phenol and analyzed the gene organization, constituting the first report characterizing catabolic genes involved in the degradation of phenol in moderate halophiles, providing an ideal model system to investigate the potential use of this group of extremophiles in the decontamination of saline environments.

## Introduction

Bioremediation of extreme environments has received little attention although many contaminated ecosystems present high or low temperatures, extremely acidic or alkaline pH, high pressures or high salinity. Extremophilic microorganisms (extremophiles) are adapted to thrive in such hostile environments constituting potential tools in the restoration of these contaminated environments [1].

The saline and hypersaline ecosystems present on Earth constitute extreme habitats that are subjected to environmental contamination with organic compounds mainly as a result of industrial activities and urban water effluents [2]. The salt concentration of these environments can vary from 3.5% (w/v) of total dissolved salts, as in seawater, to concentrations close to saturation (35%, w/v). Moreover, different industries such as the petroleum refineries generate a huge amount of oily and saline residual waters (oily brines, production waters) with salinities up to 10% (w/v) after separation of crude oil from reservoir water. The main contaminants in these production waters are aromatic and hydrocarbons, including phenolic compounds [1], [2], [3].

Conventional microorganisms are unable to operate efficiently at salinities above that of seawater and their capacity to degrade contaminants is easily lost after exposure to saline conditions [4], [5]. Thus, in the biological treatment of industrial hypersaline wastewaters and in the bioremediation of polluted hypersaline environments it is necessary to use the metabolic potential of halophilic microorganisms. The degradation or transformation of organic pollutants by halophilic and halotolerant microorganisms has not been studied in a systematic way, and only a few studies have reported the biotechnological potential of this group of extremophiles in the decontamination of saline environments [6], [7], [8], [9], [10], [11], [12]. Among halophilic microorganisms, moderate halophiles (optimal growth at 7.5–10% w/v NaCl) constitute the most versatile group to be used in the biological decontamination processes [13].

Most of the information on pollutant degradation in saline environments is regarding ecological studies performed in contaminated saline habitats, however scarce information is available concerning the prevalence of the catabolic pathways involved in the degradation of organic compounds in moderate halophiles. In fact, to our knowledge, except partial sequences of catechol 2,3-dioxygenase genes obtained from isolates in hypersaline lakes [14] and the characterization of genes encoding benzoate and *p*-hydroxybenzoate degradation in *Chromohalobacter* sp. HS-2 [15], no reports at molecular level are available on the characterization of aromatic-degrading genes in moderate halophiles.

In an ecological study in hypersaline environments contaminated with aromatic compounds to isolate moderate halophiles that could be useful in the development and optimization of bioremediation processes, an important group of 11 strains with the ability to degradate aromatic compounds, including phenolic compounds were characterized, showing a great relatedness to the genus *Halomonas* [16]. A new species named *Halomonas organivorans* was proposed and it is highlighted for its ability to utilize organic compounds (considered as pollutants) as the sole source of carbon and energy [17]. Although the complete mineralization of phenol and the catabolic pathways have been described in several bacteria [18], [19], [20], this information is lacking in halophilic bacteria.

In non-halophilic bacteria, a number of aerobic biodegradation pathways of aromatic compounds like phenol converge into catechol ring cleavage [21]. The scission of the aromatic ring is catalyzed by dioxygenases and occurs through *ortho-* or *meta-* cleavage that is followed by further steps of the catabolic pathway, which finally convert the produced metabolites into intermediates of the citrate cycle.

In the present work, we focused on the cloning and characterization of the genes involved in the metabolism of phenol and benzoate in the moderately halophilic bacterium *Halomonas organivorans* due to the importance of the halophiles in the development of new biological strategies for the restoration of hypersaline environments and saline wastewaters generated by industries.

## Results

### Isolation and cloning of *cat* genes from *H. organivorans*

In order to isolate the genes responsible of the catabolism of phenolic compounds in *H. organivorans*, a gene library was constructed using the vector pEZ seq according to the method described in Material and Methods. 1,2-CTD constitutes a key enzyme in the catechol branch of the β-ketoadipate pathway. Thus, to screen the library, degenerate primers were designed based on the conserved sequences of 1,2-CTD genes from several degrading bacteria. To amplify by PCR a conserved internal fragment of this 1,2-CTD encoding gene, we used total DNA of *H. organivorans* and the degenerate primers cat1 and cat3 (Material and Methods). A PCR fragment of 412 bp was amplified and subsequent cloning and sequencing revealed homology to the published nucleotide sequences of the 1,2-CTD genes from *Chromohalobacter* sp. HS-2, *Pseudomonas* sp. MT1 and *Pseudomonas fluorescens* SBW24 (79%, 75% and 72% identity, respectively). In the library screening, using the amplified 412 bp PCR fragment as probe, four *E. coli* positive clones were isolated and designated A1, D14, H7 and P20. These clones were analyzed showing plasmids with overlapping regions. The plasmids pHA1 and pHD14 (5,555 and 3,971 bp insert fragments, respectively) were selected for sequencing and further characterization.

### Nucleotide sequences of the *cat* genes and flanking regions

After sequencing the inserts of plasmids pHA1 and pHD14, a region of 4,265 bp was analyzed finding 6 open reading frames (ORFs). According to sequence similarity searches, these open reading frames correspond to *cat* and *ben* genes. BLAST search indicated high similarity to the genes *cat*A, *cat*B, *cat*C and the divergently oriented *cat*R gene coding for 1,2-CTD, *cis,cis*-muconate cycloisomerase, muconolactone delta-isomerase and a LysR-type transcriptional regulator, respectively. Downstream the *cat* genes, two genes *ben*A and *ben*B were identified, coding for the large and small subunits of a benzoate 1,2-dioxygenase, respectively.

### Analysis of the catabolic genes in *H. organivorans*

**Figure 1:**
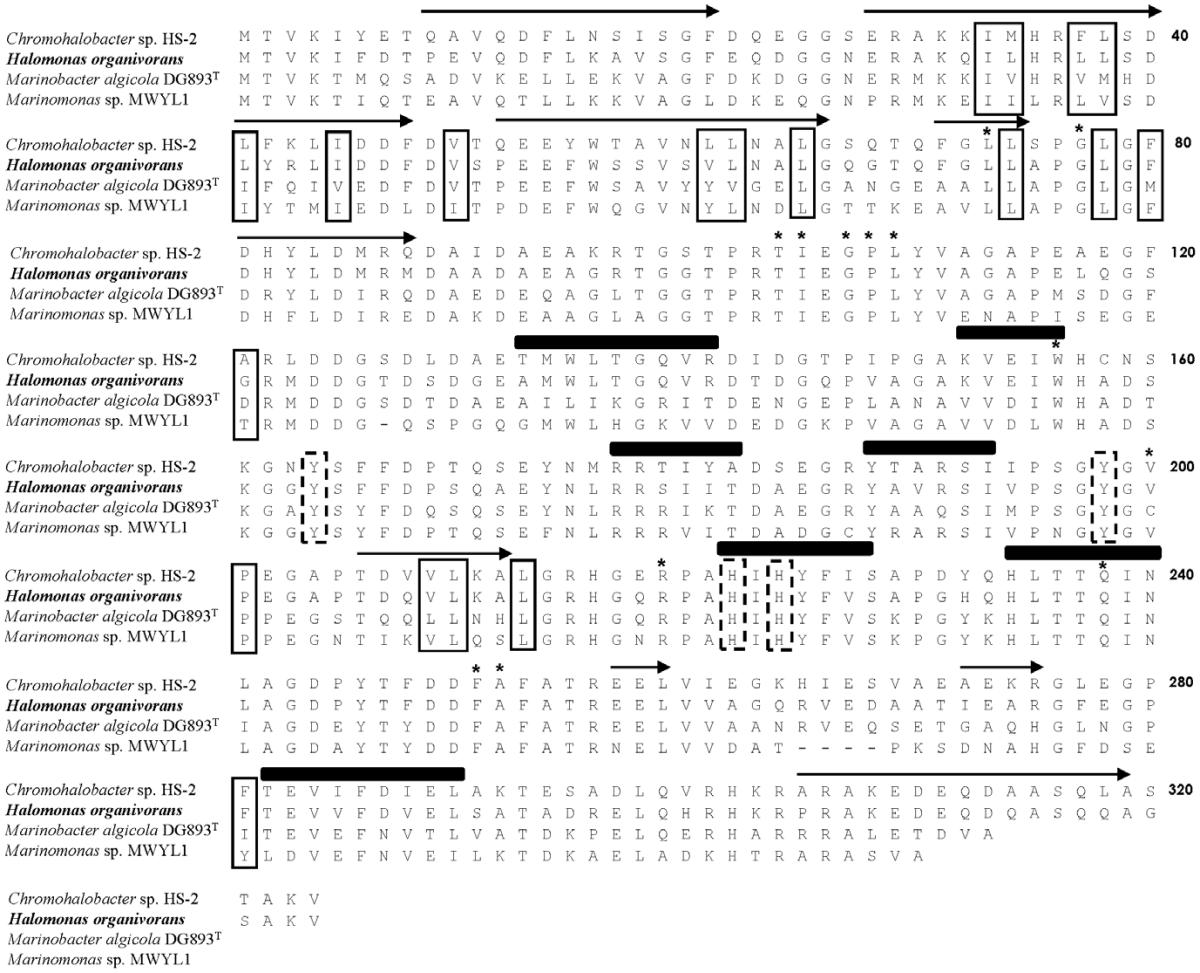
Sequence alignment of catechol 1,2-dioxygenase from *Halomonas organivorans* and related catechol 1,2-dioxygenases from halophilic bacteria. Residues in boxes show the lipid-binding sites. Residues with asterisk show the active sites. Residues in discontinuous boxes indicate the Fe-ligands. Helices are labeled with arrows and strands with black sticks.

*cat*A encodes a protein of 324 amino acid residues with a calculated molecular weight of 35.38 kDa. Sequence analysis using BLAST revealed that *cat*A gene codes for a 1,2-CTD showing the highest similarity to the 1,2-CTD from *Chromohalobacter* sp. HS2, *Marinobacter algicola* DSM 16394^T^ and *Marinobacter* sp. ELB17 (87%, 78% and 75%, respectively). Analysis of the amino acid sequences of the 1,2-CTD from *H. organivorans* reported the presence of conserved domains including the active sites, Fe-ligands and lipid-binding sites as highlighted in Figure 1. Three active sites (Leu 73, Ile 105 and Gly 107) have been identified in all known catecholic intradiol dioxygenases. Moreover, Ala 77 is a position shown to be conserved in nearly all the 1,2-CTD described to be involved in the connection between Helices D and E of the protein [22]. However, in the 1,2-CTD of *H. organivorans* and in the other halophilic strains, Ala 77 is not conserved being replaced by a residue of Gly. Cys 200 is also a well conserved position in the 1,2-CTD of degrading bacteria, described as a critical residue involved in the formation of a pocket into which other residue rotates upon binding substrate. However, as shown in Figure 1 in the four aligned 1,2-CTD only in *Marinobacter algicola* DG893^T^ Cys 200 is well conserved, being replaced by Val in the enzymes from the other halophilic degrading bacteria analyzed. It is known that the replacements in the position of amino acids determine the folding of helices and sheets. With respect to conserved domains of Fe-ligands, in the 1,2-CTD from *H. organivorans*, the non-heme Fe is supposed to be ligated by two tyrosines (Tyr 164 and Tyr 198) and two histidines (His 222 and His 224) as in other intradiol dioxygenases studied. The amino acid sequence of 1,2-CTD of *H. organivorans* evidenced also the presence of lipid-binding sites (data not shown).

*cat*B codes for a *cis,cis*-muconate cycloisomerase (89% similarity to the deduced amino acid sequence of the *cat*B gene product of *Chromohalobacter* sp. HS-2 and 82% similarity to the *cat*B gene product of *Marinobacter algicola* DSM 16394^T^) and *cat*C codes for a muconolactone delta-isomerase (91% similarity to the deduced amino acid sequence of the *cat*C gene product of *Chromohalobacter* sp. HS-2 and 89% similarity to *cat*C gene product of *Marinobacter algicola* DSM 16394^T^). Upstream of the *cat*BCA gene cluster, an incomplete ORF designated *cat*R is transcribed divergently and encodes a LysR-type transcriptional regulator with high similarity (75%) to *cat*R regulators of γ-Proteobacteria (*Chromohalobacter* sp. HS-2 and *Marinobacter algicola* DG893). *ben*A codes for the large subunit of a benzoate 1,2-dioxygenase and showed significant protein similarity (94%, 84% and 83%) to those of Gram-negative benzoate-degrading bacteria, belonging to the halophilic species *Chromohalobacter* sp. HS-2, *Marinobacter algicola* DSM 16394^T^ and *Marinobacter* sp. ELB17, respectively. *ben*B codes for the small subunit of a benzoate 1,2-dioxygenase, showing 90% similarity to the *ben*B gene product of *Marinobacter algicola* DSM 16394^T^, 88% similarity to that of *Paracoccus denitrificans* PD1222 and 83% similarity to the *ben*B gene product of *Chromohalobacter* sp. HS-2.

### Organization of *cat* and *ben* genes of *H. organivorans* and comparison with equivalent catabolic genes from other degrading bacteria

**Figure 2:**
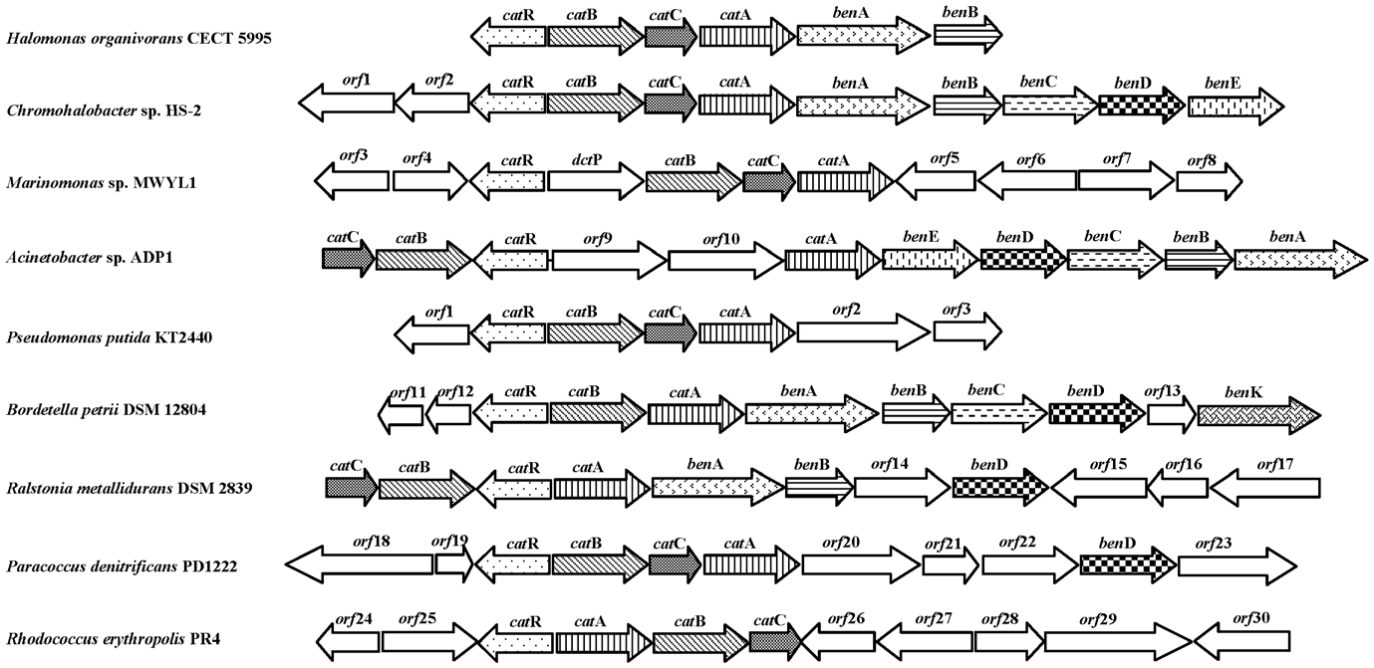
Comparative analysis of *cat* and *ben* genes from *H. organivorans* and other strains. The direction of transcription is indicated by the arrowheads. *cat*A, catechol 1,2-dioxygenase; *cat*B, *cis,cis*-muconate cycloisomerase; *cat*C, muconolactone delta-isomerase; *cat*R, lysR-type transcriptional regulator; *ben*A, benzoate 1,2-dioxygenase large subunit; *ben*B, benzoate 1,2-dioxygenase small subunit; *ben*C, benzoate 1,2-dioxygenase reductase subunit; *ben*D, 1,6-dihydroxy cyclohexe-2,4-diene-1-carboxylate dehydrogenase; *ben*E, MFS family benzoate membrane transporter; *ben*K, benzoate transporter protein.

In *H. organivorans*, the genes responsible for catechol degradation proved to be organized in the *cat*BCA operon with the *cat*R gene located upstream of *cat*B gene in the opposite orientation. The *ben*AB genes in this bacterium are contiguous to this cat-operon. The gene organization of the cat and ben clusters analyzed in *H. organivorans* was compared with that of other equivalent clusters in a number of selected bacteria (Figure 2). The organization of the cat gene cluster *cat*BCA was similar to that of the catabolic genes identified in *Chromohalobacter* sp. HS-2 [15], *Marinomonas* sp. MWYL1 [GenBank:NC_009654], *Paracoccus denitrificans* PD1222 [GenBank:NC_008686] and *Pseudomonas putida* KT2440 [23] (Figure 2). In the phylum Actinobacteria as the species *Rhodococcus erythropolis* PR4, the organization of these genes is *cat*ABC that forms a regulatory unit responsible for the degradation of catechol to β-ketoadipate enol-lactone [23] and similar homologous genes were found in *Rhodococcus opacus* 1CP [24]. In *Pseudomonas aeruginosa* PAO1c the organization described is *cat*CBA [25].

### Phylogenetic analysis based on the 16S rRNA and *cat*A genes from *H. organivorans* and other degrading bacteria

**Figure 3:**
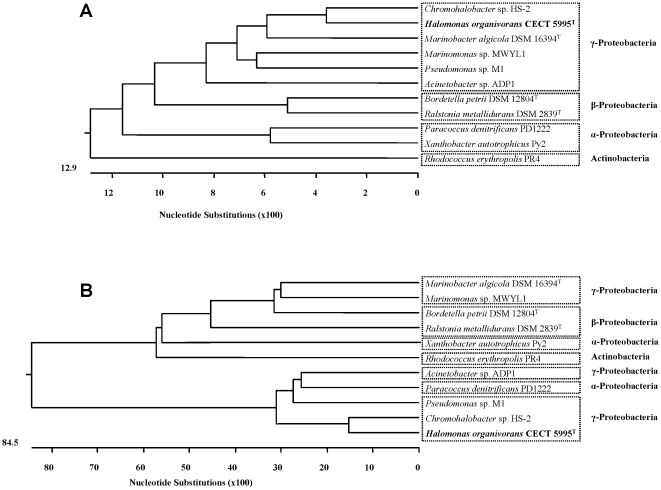
16S rRNA and *cat*A gene-based phylogenic trees. (**A**) Phylogenetic tree based on the analysis of nucleotide sequences of c. 1,500 bp of 16S rRNA gene from *Halomonas organivorans* and other degrading bacteria; (**B**) Phylogenetic tree based on the comparison of c. 840 bp of *cat*A gene from *Halomonas organivorans* and other *cat*A genes from related degrading bacteria. The trees are constructed using the software DNAstar Lasergene and the ClustalW method was used for the alignment. Branch lengths are proportional to the interfered phylogenetic distances.

16S rRNA and *cat*A gene-based phylogenies were established among *H. organivorans* and the most related degrading bacteria. For that, ten sequences available in the databases corresponding to 16S rRNA and *cat*A genes were selected, including microorganisms belonging to *α*, β and γ-Proteobacteria and Actinobacteria phyla (described in Material and Methods) in order to be aligned and construct the respective phylogenetic trees (Figure 3). *cat*A gene-based phylogenetic tree exhibited two main branches with sequences of different phyla spread along them and showing no correlation with the 16S rRNA gene-based tree that exhibited the expected topology congruent with the classical phylogenetic affiliation in which Proteobacteria grouped in different clusters (*α*, β and γ) and the representative Actinobacteria *R. erythropolis* appeared as outgroup.

### Phenol and benzoate-mediated induction of the degradative genes

**Figure 4:**
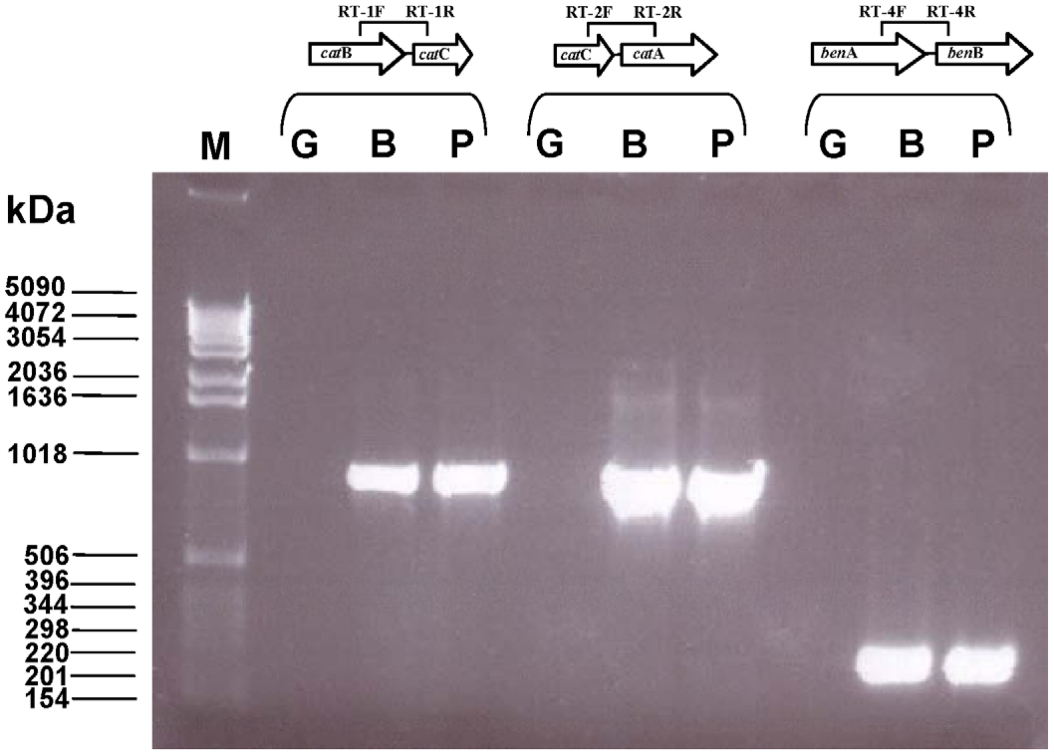
Results of RT-PCR. The PCR-products were amplified from cDNA template to prove the induction of the *cat* and *ben* genes in presence of glucose (G), benzoate (B) and phenol (P). *cat*B/*cat*C (881 bp); *cat*C/catA (866 bp); *ben*A/*ben*B (197 bp).

In order to know if the characterized degradative genes are specifically induced and expressed in presence of phenol and benzoate, we carried out reverse transcription. RT-PCR assays with primers based on the intergenic-region of the genes were performed using total RNA extracted from cells of *H. organivorans*. For this experiment, *H. organivorans* was grown in saline minimal medium using phenol and benzoate as the sole carbon source as described in detail in Material and Methods. In order to confirm that the degradative genes are induced by the two organic compounds, a control culture with glucose was carried out. Oligonucleotide primers were designed to generate specific PCR products of the gene clusters. Three products of the expected sizes were amplified with the primer pairs spanning the borders of *cat*B-*cat*C (881 bp), *cat*C-*cat*A (866 bp) and *ben*A-*ben*B (197 bp). As shown in Figure 4, all of the primer sets generated the RT-PCR products of the expected sizes on the DNA from the phenol and benzoate-grown cells of *H. organivorans*. However, no signals were obtained with cells grown in presence of glucose. Results of RT-PCR using the primer sets *cat*B-*cat*C and *cat*C-*cat*A showed that the *cat* genes are induced by phenol and benzoate and they are co-transcribed in one single operon. Results obtained with the primer set *ben*A-*ben*B demonstrated the induction of *ben* genes in the presence of phenol and benzoate, showing also the co-transcription of both genes (*ben*AB).

### Activity of catechol 1,2-dioxygenase

The induction of the key enzyme 1,2-CTD by different organic compounds in *H. organivorans* was studied in a previous work by García et al. [17]. To confirm the functionality of the characterized *cat*A gene from *H. organivorans* coding for the 1,2-CTD, a specific enzymatic assay was performed. The activity of 1,2-CTD was measured in cell-free extract from cells of *E. coli* H7 clone grown in the minimal medium M63 additioned of glucose, phenol and benzoic acid (Table 1). 1,2-CTD activity was induced with phenol and benzoic acid, although a lower activity was detected in cell-free extract from cells grown with benzoic acid. In contrast, when glucose was added to the minimal medium, no 1,2-CTD activity was detected (Table 1).

**Table 1:** Activity of catechol 1,2-dioxygenase in extracts of the clone *E. coli* H7 cells grown in various substrates.

Substrate	Specific activity^a^ (U/mg protein)
**Glucose**	0.001
**Phenol**	0.520
**Benzoic acid**	0.287

a Averages of three independent measurements are shown. Standard deviation did not exceed 22% in any data set.

### Nucleotide sequence accession number

The degradative nucleotide sequences reported in this paper have been submitted to the EMBL/GenBank/DDBJ database and assigned accession numbers FN997643-FN997647.

## Discussion

During past decades, much research has been done on elucidating the metabolism of different organic compounds in non-halophilic bacteria like *Pseudomonas*, a model genus in biodegradation studies. However, scarce information is available about the catabolism of organic compounds in halophiles [2], [7], [8], [9], [10], [11], [12].

The presence of the catechol branch (cat) and the protocatechuate branch (pca) of the β-ketoadipate pathway has been described in *H. organivorans* [17]. In different non-halophilic bacteria such as *Pseudomonas* or *Acinetobacter*, catechol is the key intermediate of the catechol branch that it is converted into *cis, cis*-muconate through dioxygenation and cleavage of the benzene ring by a 1,2-CTD [17], [22], [26], [27], [28]. *Ortho*-cleavage pathway is frequently chromosomally encoded and is widely distributed in soil bacteria and fungi, constituting the major pathway for aromatic compounds catabolism in these organisms [23], [29]. The *cat* genes are usually organized in a single cluster [29]. We report the archetype *cat*RBCA gene cluster in the moderately halophilic bacterium *H. organivorans* comprising genes encoding a *cis,cis*-muconate cycloisomerase, muconolactone delta-isomerase and 1,2-CTD, showing the *cat*R regulatory gen. Contiguous to the cat operon, the *ben* genes are present (Figure 2). Although the ben and cat gene clusters are distantly located in the genome of some bacteria as *Pseudomonas putida* KT2440 [23], in the genome of *H. organivorans* are contiguous, finding similar organization in *Chromohalobacter* sp. HS-2, *Acinetobacter* sp. ADP1, *Bordetella petrii* DSM 12804, *Ralstonia metallidurans* DSM 2839 and *Pseudomonas fluorescens* [15], [23], [30]. Moreover, ben and cat gene clusters have been found closely linked in *Pseudomonas aeruginosa* and *Burkholderia pseudomallei* [23]. By analogy to the cat gene cluster described in *Chromohalobacter* sp. HS-2 and others, *cat*R (Lys-type regulatory protein) might activate the expression of *cat*BCA genes in *H. organivorans* in response to the inducer *cis,cis*-muconate [15].

Phenol is one of the most prevalent forms of chemical pollutants, being toxic even at low concentrations. The presence of phenolic compounds in industrial wastewaters released by oil refineries, coke conversion, pharmaceutical and resin manufacturing plants poses significant risk to aquatic biota [3]. Several methods can be used for phenol degradation, such as advanced oxidation processes (AOPs) and/or biological processes. To develop biological treatments, the degradation of phenol has been extensively studied in non-halophilic bacteria and different catabolic routes have been described for this compound [31], [32]. However, biodegradation processes are difficult to perform under saline conditions [1], [13] although saline wastewaters containing phenolic compounds are frequent in refineries and various chemical industries. Thus, approaches using halophilic organisms, which are adapted to live in such saline conditions, to remove phenol under a wide range of salinities constitute an interesting alternative. We have demonstrated that the expression of the characterized *cat* and *ben* genes from *H. organivorans* is induced in presence of phenol and benzoic acid (Figure 4; Table 1). To our knowledge, this is the first report describing catabolic genes involved in the degradation of phenol in moderate halophiles, providing additional data to support the establishment of cost effective methods to remediate phenolic saline wastewater effluents.

The deduced amino acid sequence of *cat*A coding for the 1,2-CTD in *H. organivorans*, showed the presence of active residues identical to those found in the catechol dioxygenases that have been previously structurally studied. However, two residues, Gly 77 and Val 200, are only well conserved in the 1,2-CTD from the halophiles studied, suggesting an implication in the halophilic character. The results based on the search of Fe-ligands suggest that the 1,2-CTD from *H. organivorans* could present a trigonal bipyramidal geometry identical to other intradiol dioxygenases whose structures have been studied by crystallographic assays [22]. On the other hand, Vetting et al. [33] described that the dimeric catechol intradiol dioxygenases are lipid-binding proteins. In this sense, the study of the structure of 1,2-CTD from *Pseudomonas arvilla* revealed the presence of peaks with masses consistent with lipids [22]. The presence of lipid-binding sites in the amino acid sequence of *H. organivorans* as shown in the Figure 1 supports this general lipid-binding characteristic, although the physiological function for the bound of lipids in this family of enzymes is unknown.

The use of the *cat*A gene as a molecular phylogenetic marker has been previously described [34], [35]. Thus, in this work we performed a comparison between the *cat*A and 16S rRNA genes based phylogenies. However, the phylogenetic trees constructed based on the 16S rRNA and *cat*A genes from representative microorganisms retrieved from the Genbank database revealed no correlation among them. Results obtained with sequences of *cat*A gene showed a distribution of the branches with two main groups in which there is no grouping of species in the well-known subgroups of Proteobateria phyla. Therefore, we can conclude that 16S rRNA and *cat*A genes evolved independently. There are evidences indicating that horizontal gene transfer is a common mechanism for introducing catabolic pathways in bacterial genomes [36].

The usefulness of *cat*A sequence to differentiate microorganisms at the interspecies level, complementing the overall phylogenetic information has been previously proposed [34], [35] and the implication of both genes (16S rRNA and *cat*A) in the industrial monitoring of the bacterial degraders is shown in this work. On the other hand, El Azhari et al. [37] indicated the absence of congruence between data from 16SrRNA and *cat*A gene sequences which suggest horizontal gene transfer of the *cat*A gene cluster.

## Materials and Methods

### Bacterial strains, plasmids, media and culture conditions

*H. organivorans* was routinely grown in a saline medium (SW) with a final concentration of 10% (w/v) total salts (SW-10) supplemented with 0.5% (w/v) yeast extract (Difco) [38]. RT-PCR analysis was carried out using cultures of *H. organivorans* in the minimal medium M63 [39] containing 5 mM benzoic acid, glucose and 2.5 mM phenol as the sole carbon source and a final concentration of 10% (w/v) total salts (SW-10). The pH of the media was adjusted to 7.2 with KOH. *Escherichia coli* DH5α was used as the host for routine subcloning and was grown in Luria-Bertani (LB) medium [40]. Solid media contained 1.8% (w/v) agar (Difco). When appropriate, the antibiotics ampicilin (30 µg ml^−1^) and kanamycin (50 µg ml^−1^) were added to the media. The pGEM-T easy vector (Promega) (ampicilin resistant) was used for subcloning of the PCR products and enzyme-restricted DNA fragments. The host cloning vector pEZ seq (Lucigen) (kanamycin resistant) was used to construct a genomic library of *H. organivorans*. All restriction and modification enzymes (Amersham Biosciences) were used as recommended by manufacturers. Primers were synthesized and purchased from MWG biotech. The standard molecular biology protocols followed were described by Sambrook and Rusell [40].

### DNA manipulations

DNA manipulations and isolation of plasmid DNA were performed by standard procedures [40]. Southern blot analyses were carried out by using digoxigenin-labelled probes according to the instructions of the manufacturer (Roche).

### Amplification of 1,2-CTD gene

To detect the presence of catabolic genes encoding key enzymes of the metabolic pathways in *H. organivorans*, PCR amplification was performed using degenerate primers for the 1,2-CTD gene. The primers for the amplification were the following: cat1 (5′-ACCATCGARGGYCCSCTSTAY-3′) and cat3 (5′-GTTRATCTGGGTGGTSAG-3′) designed from two conserved regions of different 1,2-CTD, spanning residues 100–106 and 232–237 from *Acinetobacter radioresistens* (Accession No. AAG16896). Each PCR mixture contained: 5 µl of 10× reaction buffer (Promega), 2.5 µl of 25 mM MgCl_2_, 8 µl of dNTPs (200 µM each), 50 pmol of the appropriate primers, 0.25 µl of *Taq* polymerase (Promega) and sterile distilled water to adjust the total volume to 50 µl. The PCR conditions for the amplification of 1,2-CTD encoding gene consisted of an initial cycle of 5 min at 95°C, followed by 35 cycles of denaturation at 94°C for 1 min, annealing at 50°C for 1 min, and extension at 72°C for 1 min. The amplified products were analyzed on 1% (w/v) agarose gels stained with ethidium bromide and photographed with UV illumination.

### Enzyme analysis

The clone *E. coli* H7, containing the gene cluster *cat*RBCA of *H. organivorans* involved in catechol degradation was grown in the minimal saline medium M63 additioned with phenol (2.5 mM), benzoic acid and glucose (5 mM) as the sole carbon source and energy. The cells were harvested by centrifugation and the cell pellets were resuspended in a volume of breaking buffer (50 mM Tris HCl, pH 7.5, 1 M glycerol, 5 mM ammonium sulphate, 2.5 mM MgCl_2_, 1 mM EDTA, 1 mM DTT) and sonicated using a tip sonicator; the suspensions were centrifuged at 11000× g for 3 min at 4°C. The clear supernatants obtained were used for enzymatic assays. Activity of 1,2-CTD was measured spectrophotometrically at 25°C following the formation of *cis,cis*-muconic acid by measuring the absorbance at 260 nm [41]. One unit of enzyme activity was defined as the amount of enzyme to form 1 µmol/L product per min. Specific activity of the enzyme was defined as units per mg of protein. The protein concentration was determined by the method of Bradford, using BSA as the standard [42].

### Phylogenetic analysis

16S rRNA nucleotide sequences (c. 1500 bp corresponding to positions 1–1500 of the 16S rRNA gene from *Escherichia coli*) and catA nucleotide sequences (c. 840 bp) from γ, β, *α*-Proteobacteria and Actinobacteria phyla were retrieved from databases with the following accession numbers: *Chromohalobacter* sp. HS-2 [GenBank:EU045305]/[GenBank:EU155151, positions 15199 to 16173], *Marinobacter algicola* DSM 16394^T^ [GenBank:AJ294359]/[GenBank:NZ_ABCP01000005, positions 73804 to 74094], *Marinomonas* sp. MWYL1 [GenBank:EF094505]/[GenBank:NC_009654, positions 3434825 to 3435742], *Pseudomonas* sp. MT1 [GenBank:DQ026293]/[GenBank:DQ026294, positions 7076 to 7999], *Acinetobacter* sp. ADP1 [GenBank:AY289925]/[GenBank:NC_005966, positions 1439848 to 1440783], *Bordetella petrii* DSM 12804 [GenBank:NC_010170, positions 3503937 to 3502452]/[GenBank:NC_010170, positions 3957681 to 3958436], *Ralstonia metallidurans* DSM 2839 [GenBank:NC_007973, positions 3617953 to 3616427]/[GenBank:NC_007973, positions 1543053 to 1543976], *Paracoccus denitrificans* PD1222 [GenBank:NC_008687, positions 141979 to 143453]/[GenBank:NC_008687, positions 1147377 to 1148315], *Xanthobacter autotrophicus* Py2 [GenBank:NC_009720, positions 2150387 to 2151864]/[GenBank:NC_009720, positions 4830443 to 4831357] and *Rhodococcus erythropolis* PR4 [GenBank:NC_012490, positions 4279557 to 4281076]/[GenBank:NC_012490, positions 5872478 to 5873317]. These sequences were aligned to the 16S rRNA and *cat*A nucleotide sequences from *Halomonas organivorans* using the ClustalW software (ver. 1.81) with BLOSUM62 matrix and manually edited. The phylogenetic tree was inferred using the Neighbour-joining method [43].

### Construction and screening of a gene library of *H. organivorans* in *E. coli*

A *H. organivorans* gene bank was constructed in the vector pEZ seq (Lucigen). *H. organivorans* genomic DNA was partially digested with *Bam*HI and DNA fragments were separated and cloned into the *Bam*HI site of pEZ seq. Aliquots of the reaction mixture were used to transform cells of *E. coli* DH10B (Invitrogen) that were then plated onto LB agar plates containing kanamycine to select recombinant clones. Randomly selected clones were found to contain DNA inserts ranging from 5 to 10 Kb in size. Library screening was performed on the basis of direct colony-Southern blot hybridization using a digoxigenin labelled probe and a specific detection kit (Boehringer Mannheim). As probe, a 412 bp fragment of the *cat*A gene was amplified using the specific primers cat1 and cat3.

### RNA isolation, preparation of cDNA and RT-PCR analysis

5 ml of exponential phase cultures of *H. organivorans* (OD_600_ of 0.5–0.7) growth in minimal medium M63 containing benzoic acid, glucose (5 mM) or phenol (2.5 mM) were harvested for RNA isolation. RNA was extracted using the High Pure RNA Isolation Kit (Roche) and eluted in 40 µl of RNase-free water. Contaminating DNA in the RNA preparation was treated twice using DNasel (Amersham Biosciences), according the manufacturer instructions. RNA samples were stored at −80°C until use. Precautions were taken during the experiment to create and maintain an RNase-free environment. Retrotranscription was performed with 5 µg of the extracted RNA in a total volume of 20 µl using a Transcriptor First Strand cDNA Synthesis Kit with random hexamer primers (Roche). The resulting cDNA was stored at −20°C for PCR experiments. The PCR reactions were performed using 30 cycles of 45 s at 96°C, 30 s at 60°C and 45 s at 72°C. Three pairs of primers were designed on the basis of the regions *cat*B-*cat*C, *cat*C-*cat*A and *ben*A-*ben*B respectively: RT-1F/RT-1R (GGCGATCGGGCAAGCGTCAG/GCTGTCGTCGTCACGGATCG), RT-2F/RT-2R (GAAACTGCCGCCGGAGATGC/CTCGGCGTCGGTGATGATCG) and RT-4F/RT-4R (GGGGCTCTCTACGTGCTGCAC/CAGGTCAGCCATTCGTCCCAC) (expected sizes 881 bp, 866 bp and 197 bp). Positive and negative controls were included in all assays.

### Computer sequence analysis

Database searches were performed using BlastX program of the National Center for Biotechnology Information (NCBI) (http://www.ncbi.nlm.nih.gov/Blast). Open reading frames (ORFs) were identified using the program ORF Finder (http://www.ncbi.nlm.nih.gov/gorf/gorf.htlm). Protein and nucleotide alignments were performed by using the ClustalW program (version 1.81) of EBI (European Bioinformatics Institute). Analysis of proteins was carried out using programs from the ExPASy Proteomics Server (Expert Protein Analysis System) including PROTPARAM (http://www.expasy.ch/tools/protparam.html) and the search for conserved domains was performed with CDD (http://www.ncbi.nlm.nih.gov/gorf/Structure/cdd/cdd.shtlm).
